# Genomic-Based Restriction Enzyme Selection for Specific Detection of *Piscirickettsia salmonis* by 16S rDNA PCR-RFLP

**DOI:** 10.3389/fmicb.2016.00643

**Published:** 2016-05-09

**Authors:** Dinka Mandakovic, Benjamín Glasner, Jonathan Maldonado, Pamela Aravena, Mauricio González, Verónica Cambiazo, Rodrigo Pulgar

**Affiliations:** ^1^Laboratorio de Bioinformática y Expresión Génica, Instituto de Nutrición y Tecnología de los Alimentos, Universidad de ChileSantiago, Chile; ^2^Fondap Center for Genoma RegulationSantiago, Chile; ^3^Laboratorio de Genómica Aplicada, Instituto de Nutrición y Tecnología de los Alimentos, Universidad de ChileSantiago, Chile

**Keywords:** computer-simulation analyses, *Piscirickettsia salmonis*, 16S rDNA, PCR-RFLP, restriction enzymes

## Abstract

The gram negative facultative bacterium *P. salmonis* is the etiological agent of Salmonid Rickettsial Septicaemia (SRS), a severe disease that causes important economic losses in the global salmon farmer industry. Despite efforts to control this disease, the high frequency of new epizootic events indicate that the vaccine and antibiotics treatments have limited effectiveness, therefore the preventive and diagnostic approaches must be improved. A comparison of several methodologies for SRS diagnostic indicate differences in their specificity and its capacity to detect other bacteria coexisting with *P. salmonis* in culture media (contamination) and fish samples (coinfection), aspects relevant for research, vaccine development and clinical diagnostic. By computer-simulation analyses, we identified a group of restriction enzymes that generate unique *P. salmonis* 16S rDNA band patterns, distinguishable from all other bacteria. From this information, we designed and developed a PCR-RFLP (Polymerase Chain Reaction—Restriction Fragment Length Polymorphism) assay, which was validated using 16S rDNA universal primers and restriction enzyme *Pma*CI for the amplification and digestion, respectively. Experimental validation was performed by comparing the restriction pattern of *P. salmonis* with the restriction patterns generated by bacteria that cohabit with *P. salmonis* (fish bacterial isolates and culture media contaminants). Our results indicate that the restriction enzyme selection pipeline was suitable to design a more specific, sensible, faster and cheaper assay than the currently used *P. salmonis* detection methodologies.

## Introduction

Salmonid Rickettsial Septicaemia (SRS) is a severe disease that causes important economic losses in the global salmon farmer industry (Rozas and Enríquez, 2014). SRS covers a wide geographic range and its outbreaks have been reported in Canada, Norway and Ireland. However, mortalities have not been as high as those recorded in Chile (Reid et al., 2004), where economic losses exceed the $100 million US per year (Lhorente et al., 2014). *Piscirickettsia salmonis*, its etiological agent, is a gram negative intracellular facultative bacterium (Cvitanich et al., 1991; Gómez et al., 2009) that has the ability to infect, replicate and propagate in several fish cellular lines, including salmonid monocytes/macrophages (McCarthy et al., 2008). It has been recognized as the major fish pathogen for over 20 years in Chile, reason why the Chilean Fisheries and Aquaculture Center (Sernapesca) have implemented a Specific Health Surveillance Program for SRS (www.sernapesca.cl)^1^ that consists in periodic diagnostic tests to identify the presence of *P. salmonis* in fish from farm centers. Nevertheless, the high frequency of new epizootic events indicate that the current managements, including vaccine and antibiotics treatments, have limited effectiveness and that the preventive and diagnostic approaches must be improved (Henríquez et al., 2015).

To diagnose SRS, several methods have been developed, including solid culture of *P. salmonis* coupled with Gram and Giemsa stain technique (Mauel et al., 2008; Mikalsen et al., 2008; Vera et al., 2012), conventional PCR (Mauël, 1996; Marshall et al., 1998) real time PCR (Corbeil et al., 2003; Karatas et al., 2008), and indirect fluorescence antibody test (Lannan et al., 1991). All of them have been demonstrated as competent methodologies to detect *P. salmonis*, but none of them can guarantee specificity nor can demonstrate the exclusive presence of the bacterium in tissue samples and culture media. Culture media used to grow *P. salmonis* are highly nutritive and non-selective, therefore they can become contaminated easily by other bacteria. In addition, *P. salmonis* is of slow growth, developing visible colonies over 4 days after culturing, and since the Gram staining only allows to distinguish general morphological aspects of bacteria, microbiological approaches to diagnose *P. salmonis* are not viable at an industrial level. On the contrary, molecular methods based on DNA amplification analyses are accurate, faster and more sensible than culture techniques, but more expensive, particularly those based in fluorescence detection (Scheler et al., 2014). Furthermore, methods centered in immunodetection and microscopy are costly and highly time consuming.

Since the design of primers, probes and antibodies for *P. salmonis* detection were developed several years ago using limited genomic information, we can hypothesize that they may not be specific enough. For this reason, and taking advantage of the fast-growing ribosomal genes sequences databases, we propose a genomic-based pipeline that selects, among restriction enzymes available, the ones capable to digest 16S rDNA gene of *P. salmonis* that generate band patterns distinguishable from all other bacteria. With this information, we developed and validated a PCR-RFLP assay that detects *P. salmonis*, bringing back the advantages of traditional molecular methods to detect *P. salmonis* in fish tissues and culture media, such as sensitivity and speed, and improving the cost and specificity.

## Materials and methods

### 16s rDNA sequences and restriction enzymes bioinformatic selection

The complete set of 16S rDNA sequences (not trimmed sequences = 5,030,478, “original 16S rDNA databases”) was retrieved from Greengenes version 13.8 (DeSantis et al., 2006), Ribosomal Database Project version 11.4 (Cole et al., 2009) and SILVA version 123 (Pruesse et al., 2007) databases, excluding sequences from chloroplast, mitochondria, eukarya and archaea (Supplementary Figure 1). To maximize the probability of finding restriction sites we selected only those sequences that contain the 16S rDNA gene universal primers 27F (5′-AGAGTTTGATCCTGGCTCAG-3′) and 1492R (5′-CGGTTACCTTGTTACGACTT-3′) (Jiang et al., 2006). Each sequence in our database was trimmed into the fragment flanked by two conserved regions corresponding to the primers sites (trimmed sequences = 568,169, “processed 16S rDNA databases”) using reads_fasta and find_adaptor scripts of the bioinformatics suit Biopieces version 0.51 (Supplementary Figures 1, 2). Then, we retrieved and manually cured all sequences of *P. salmonis* obtaining 15 non-redundant representatives of the different strains (indicated in bold with SILVA database ID in Supplementary Table 1). In order to obtain the restriction patterns, each trimmed sequence from our database was *in silico* digested using the script Restrict from EMBOSS Suit version 6.3.1 with the following parameters: snucleotide1, sitelen = 4, rformat = table, enzymes = enzymes.txt. From the 4379 sequences present in REBASE, we selected the 650 restriction enzymes that were commercially available, which were contained in “enzymes.txt.” From the 650 enzymes, 152 digest all *P. salmonis* sequences and only 65 recognized conserved restriction sites in the complete set of sequences. With the aim of selecting restriction enzymes that generate easily differentiable restriction patterns between *P. salmonis* and other bacteria, we designed a *perl script* which compares each band pattern produced by each enzyme of selected *P. salmonis* reference sequences against the band patterns produced by each enzyme when digesting all other 16S rDNA sequences from our database. If digestion of a non- *P. salmonis* 16S rDNA sequence produced a predicted band pattern that matched exactly with the reference pattern, and there are no more or less bands that would permit to differentiate the patterns, then the enzyme is marked as “bad” (*n* = 52). All enzymes that were not marked as bad (good enzymes) were manually reviewed (*n* = 13) (Supplementary Figure 1; Table 1). The intragenomic and intergenomic heterogeneity was calculated by Shannon information entropy (Sun et al., 2013) at each nucleotide position using the six copies of 16S rDNA gene of the complete *P. salmonis* genomes (Pulgar et al., 2015b) and the 15 non-redundant *P. salmonis* sequences of different strains, respectively.

**Table 1:** **Features of the thirteen selected restriction enzymes**.

**Enzyme**	**Recognition site**	**US$/Unit**	**Average pattern in non-redundant** ***P. salmonis*** **strains**									
*AjuI*	(7/12)GAANNNNNNNTTGG(11/6)	0.59	32	572	901							
*AsuI*	G/GNCC	0.06	1	126	133	179	190	279	597			
*BbvI*	GCAGC(8/12)	0.21	170	236	239	355	505					
*BsaAI*	YAC/GTR	0.06	97	281	395	732						
*BseGI*	GGATG(2/0)	0.03	27	362	518	598						
*Eco57I*	CTGAAG(16/14)	0.20	174	547	784							
*FnuDII*	CG/CG	0.06	15	52	103	106	112	116	143	184	291	383
*FokI*	GGATG(9/13)	0.06	7	362	531	605						
*HpaII*	C/CGG	0.03	11	52	76	81	130	216	444	495		
*MboII*	GAAGA(8/7)	0.21	84	94	386	414	527					
*MslI*	CAYNN/NNRTG	0.13	43	52	397	1013						
*OliI*	CACNN/NNGTG	0.13	43	397	1065							
*PmaCI*	CAC/GTG	0.03	97	281	395	732						

For the construction of the tree, we analyzed all 257 sequences named as *Piscirickettsia salmonis* 16S rDNA gene obtained from the RDP (*n* = 78), Greengenes (*n* = 12), SILVA (*n* = 94) and NCBI (*n* = 73) databases. These sequences were filtered manually, obtaining 36 non-redundant and trimmed sequences with a length alignment of 1251 bp, all which could be associated to an ID in SILVA database. A multiple alignment of the edited sequences was performed with the Muscle v3.8.31 software (Edgar, 2010) using the default parameters. MEGA v6.0 software (Tamura et al., 2013) was used to build a phylogenetic tree based on maximum-likelihood using general time reversible model of nucleotide substitution with invariant sites (Goldman, 1990). Bootstrap analysis (1000 pseudo-replicates) was used to evaluate statistical nodal support.

### Bacterial strains, media and growth conditions

*Piscirickettsia salmonis* LF-89 ATCC® VR-1361 was obtained from the ATCC culture collection on 2013. *Piscirickettsia salmonis* LF-89 and all the other strains used in this study (Table 2), were cultivated at 18°C in solid media (Mauel et al., 2008; Mikalsen et al., 2012; Vera et al., 2012) and/or liquid media with constant stirring of 100 rpm (Gómez et al., 2009; Vera et al., 2012; Yañez et al., 2012; Henríquez et al., 2013). Bacteria isolated from fish used in this work were obtained directly from environmentally infected Atlantic salmon (*Salmo salar*) from fish net-cages (Puerto Montt, Chile) as described by Fryer et al. (1992) with few modifications. Briefly, heart, spleen, kidney and gills were aseptically removed from moribund fish and immersed in MEM medium (Gibco), gently blended and inoculated directly into the previously described solid media at 18°C for 5–10 days for posterior selection of different morphological isolates. Among these isolated bacteria, major fish pathogens affecting Chilean salmon farming industry were identified. Laboratory contaminants were obtained from previously mentioned liquid and solid *P. salmonis* media, which were kept exposed for 24–96 h at different working places in our laboratory. Different morphological isolates were plated in solid media 18°C and/or cultivated at the same temperature with constant stirring (100 rpm) in liquid media.

**Table 2:** **Bacterial strains used in this study for validation of bioinformatic analyses**.

**Strain**	**Accession number**	**Category**
*Piscirickettsia salmonis* LF-89	KU204892	Isolated from fish
*Aeromonas salmonicida* INTA1	KU204881	Isolated from fish
*Shewanella frigidimarina* INTA2	KU204882	Isolated from fish
*Photobacterium phosphoreum* INTA3	KU204883	Isolated from fish
*Psychrobacter nivimaris* INTA4	KU204884	Isolated from fish
*Arthrobacter oxydans* INTA5	KU204885	Laboratory contaminant
*Staphylococcus saprophyticus* INTA6	KU204886	Laboratory contaminant
*Microbacterium lacus* INTA7	KU204887	Laboratory contaminant
*Escherichia coli* INTA8	KU204888	Laboratory contaminant
*Flavobacterium psychrophilum* INTA9	KU204889	Isolated from fish
*Renibacterium salmoninarum* INTA10	KU204890	Isolated from fish
*Vibrio anguillarum* INTA11	KU204891	Isolated from fish

### DNA extractions, 16s rDNA amplifications and sequencing

Bacterial DNA was purified from 1 mL of exponential phase growth cultures (OD_600_ ~ 0.5) or from ≤ 20 mg of *P. salmonis* infected tissue samples using the DNeasy Blood and Tissue Kit for DNA (Qiagen, California, United States). Bacterial culture samples were centrifuged for 10 min at 5000 g, supernatant was discarded and the pellet obtained was lysated according to the manufacturer's instructions. For infected tissue samples and infected embryonic cells, the standard protocols were used according to the manufacturer's instructions. For 16S rDNA PCR amplifications, primers 27F/1492R were used. 16S rDNA PCR amplifications were carried out in 25 μL volumes containing 200 ng (~4 μL) of bacterial DNA, 12.5 μL of GoTaq mix (Promega, Wisconsin, United States), 5.5 μL of nuclease free water and 1 μL of each primer (10 mM). The PCR amplification was performed in MJ research, Inc. Thermal cycling controller with the following protocol: 10 min at 95°C, 30 cycles of 95°C for 60 s, 58°C for 30 s and 72°C for 60 s, and a final extension at 72°C for 10 min. PCR products were kept at 4°C until use. Extracted bacterial DNA and PCR products were visualized in 2% (w/v) agarose gel electrophoresis in TAE buffer (1X), stained with 0.5 μg/ml final concentration of ethidium bromide, visualized in an UV transilluminator and photographed. Some samples were also visualized in TapeStation 2200 (Agilent Technologies, California, United States) using DNA ScreenTape and Agilent kit plus reagents according to the fabricant's indications. PCR products from 16S rDNA amplifications of the strains, infected tissues and infected embryonic cells used in this study were sequenced in Macrogen USA, while the identification of the species was based on the best sequence match obtained by Blast alignment with the 16S ribosomal RNA sequences (Bacteria and Archaea) available at NCBI database. Sequences of 16S rDNA gene from isolated strains were deposited in GenBank under accession numbers (KU204881-KU204892) (Table 2) and from tissues and embryonic cells infected with *P. salmonis* were deposited in GenBank under accession numbers (KX059708-KX059716).

Total DNA from tissues infected with *P. salmonis* was extracted as previously described and a PCR amplification of a portion of the 60S rDNA gene of *Salmo salar* was performed using primers Ss60SF (5′-CATTGATGATGGCACCTCAG-3′) and Ss60SR (5′-CTTGGCAACCTTCTTCTTGC-3′). The PCR amplification was performed in MJ research, Inc. Thermal cycling controller with the following protocol: initial step of 94°C for 2 min followed by 30 cycles of 94°C for 30 s, 60°C for 30 s, and 72°C for 30 s, with a final extension step at 72°C for 5 min. PCR products were kept at 4°C until use and were visualized as previously described.

### PCR-RFLP assay using *PmaCI*

Five microliter of 16S rDNA amplification product using primers 27F/1492R were digested using restriction enzyme *Pma*CI (FastDigest Eco72I, ThemoFisher, Massachusetts, United States) for 15 min at 37°C. The same enzyme from different brands and also their available isoschizomers were used to validate the assay. In order to compare the different band patterns, restriction fragments were run in a 2% (w/v) agarose gel electrophoresis in TAE buffer (1X), stained with 0.5 μg/ml final concentration of ethidium bromide, visualized in an UV transilluminator and photographed. Fragments were also visualized in TapeStation 2200 using DNA ScreenTape and Agilent kit plus reagents according to the fabricant's indications. The predicted restriction patterns of the 16S rDNA gene from all bacterial strains used in this study were visualized using NEBcutter web tool.

### Polymerase chain reaction (PCR) assays

ITS-PCR were developed as describe by Marshall et al. (1998) using the primers RTS1 (5′-TGATTTTATTGTTTA GTGAGAATGA-3′) and RTS2 (5′-AAATAACCCTAAATT AATCAAGGA-3′) and RTS1 and RTS4 (5′-ATGCACTTA TTCACTTGATCATA-3′). Nested PCR were performed as described by Corbeil and Crane (2009) using primers EubB (5′-AGAGTMGATCMT GGCTCAG-3′) and EubA (5′-AAGGAGGTGATCCANCCR CA-3′) for the first amplification and primers PS2S (5′-CTAGGA GATGAGCCCGCGTTG-3′) and PS2AS (5′-GCTACA CCTGCGAAACCACTT-3′) for the seconds amplification in MJ research, Inc. Thermal cycling controller. Real time PCR were performed as described by Karatas et al. (2008) using primers 16SRNA-F1 (5′- AGGGAGACT GCCGGTGATA-3′) and 16SRNA-R (5′-ACTACG AGGCGCTTTCTCA-3′). Taqman probe assays were performed as described by Corbeil et al. (2003) using primers F-760 (5′-TCTGGGAAGTGT GGCGATAGA-3′) and R-836 (5′-TCCCGACCT ACTCTTGTTTCATC-3′) and the 6-carboxyfluorescein (6FAM) and 6-carboxytetramethylrhodamine (TAMRA) labeled probe PS23S (5′-6FAM-TGA TAGCCCCGTACACGAAACGGCATA-TAMRA-3′) certified by an authorized diagnostic laboratory by Sernapesca (www.sernapesca.cl).

### Gram staining and indirect fluorescent antibody test (IFAT)

Gram staining was performed fixing the samples on a glass plate with heat. The staining process was made using crystal violet for 1 min, iodide for 1 min, ethanol for 30 s and safranin for 30 s exposition intervals. Between each interval, samples were washed with distilled water. Gram stained samples were captured in Nikon Eclipse Ni microscope with 100X objective using immersion oil. Indirect Fluorescent Antibody Test (IFAT) (SRS-Fluorotest indirect, GrupoBios, Santiago, Chile) was performed according to the fabricant's recommendations with some modifications. Twenty microliters of each sample were dried on a sterile cover slip at room temperature and fixed with paraformaldehyde (4%) for 10 min followed by three washes with sterile PBS solution. A 100 microliters of Oligoclonal reagent diluted 1:100 were added to each sample and incubated 30 min at room temperature in a moisture chamber. Then, samples were washed two times for 4 min with washing solution previously diluted 1:25 with distilled water. One hundred microliters of anti-IgG FITC solution diluted 1:100 and DAPI (Molecular probes) diluted 1:200 in dilution solution were added to each sample and incubated at room temperature for 30 min in a moisture chamber. Samples were washed two times with washing solution diluted 1:25 with distilled water for 4 min. Six μL of Dako fluorescence mounting medium (Agilent Technologies, California, United States) was added over the glass plate and samples were incubated at 4°C until the solution was dried. Samples were captured in Nikon Eclipse Ti confocal microscope with 60X objective using immersion oil.

### *In vitro* infections and tissue sample assay

*P. salmonis* LF-89 *in vitro* infections of *Oncorhynchus tshawytscha* CHSE-214 embrionic cells were performed as described by Fryer et al. (1992). For tissue sample assays of challenged Atlantic salmons by intraperitoneal infection with *P. salmonis*, head kidney, spleen and brain tissue samples were collected from early state (3 days post-infection) and late state (14 days post- infection) as described in Pulgar et al. (2015a). *Oncorhynchus mykiss* and *Oncorhynchus kisutch* head kidneys were obatined from fish with typical SRS signology. Non-infected cells and tissues from healthy fish were also used as experimental controls. The trials were approved by the Ethics Committee of the Institute of Nutrition and Food Technology, University of Chile. DNA extractions and PCR-RFLP assays were performed to these samples as explained previously.

## Results

### *P. salmonis* PCR-RFLP is a specific tool for bacterial detection

**Figure 1:**
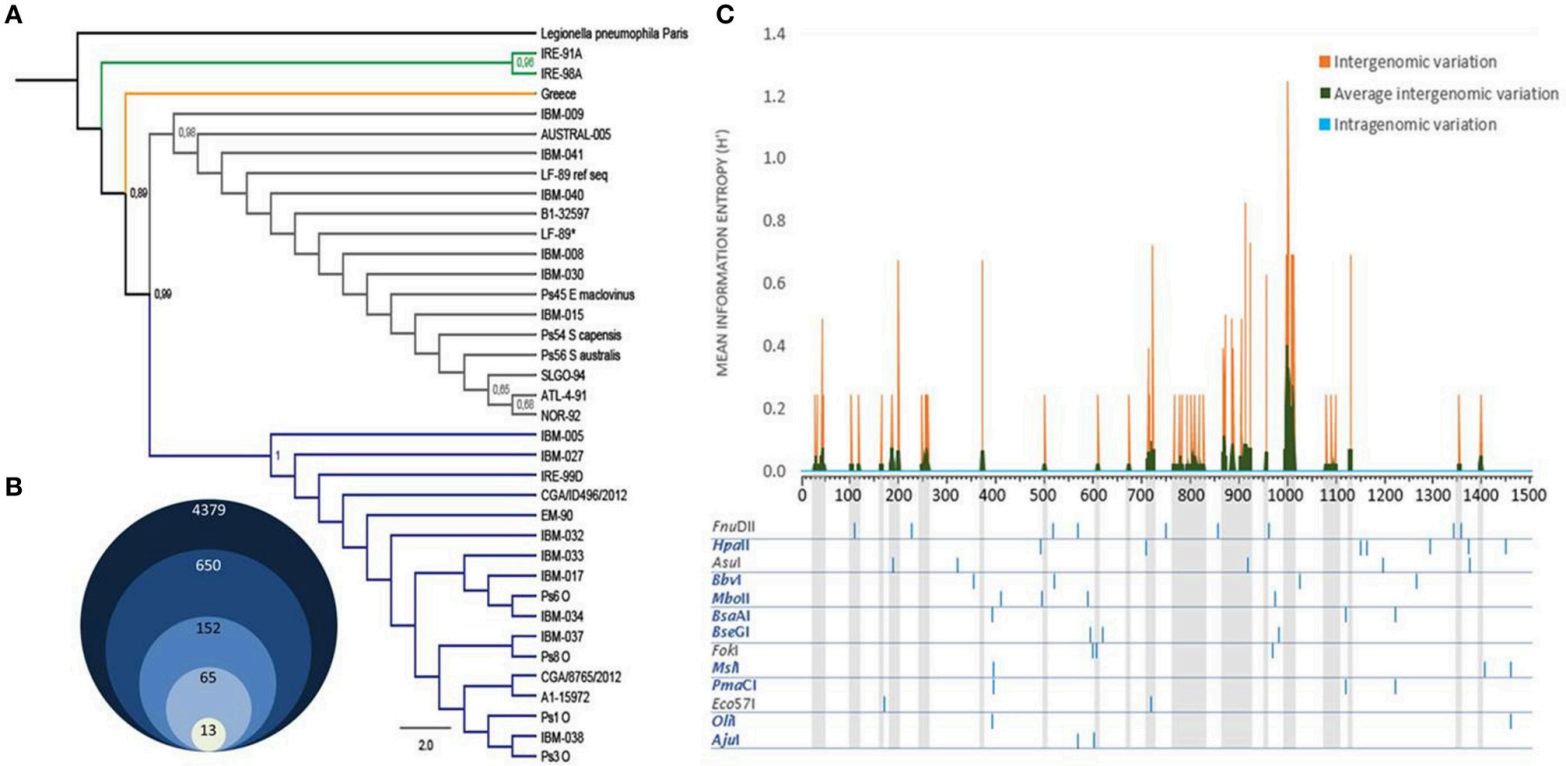
*****P. salmonis*** 16S rDNA gene characterization. (A)** Maximum-likelihood tree resulting from the analysis of 16S rDNA sequences of *P. salmonis*. Thirty six filtered sequences of 1251 bp of alignment length were used as ingroup. *Legionella pneumophila* strain Paris 16S rDNA sequence was used as outgroup (black branch). The numbers along the branches indicate bootstrap support values (only values ≤ 0.65 are shown). Green branches correspond to two Irish strains and the orange branch to one Greek strain. LF-89 ref seq represents the strain's 16S rDNA reference sequence in NCBI. LF-89^*^ represents the strain's 16S rDNA sequenced in this study (Supplementary Table S1). Blue and gray branches represent two genetically distant groups formed by the other 33 strains, including all Chilean native strains (Supplementary Table S1). **(B)** Grouped diagram showing the initial number of restriction enzymes and the selected ones used for final analyses (for restriction enzymes selection criteria see Section Materials and Methods). **(C)** Intergenomic variation rate was measured as the Shannon information entropy at each position of *P. salmonis* 16S rDNA (orange) and subsequently averaged by a 10-bp window (green). Similarly, the intragenomic variation was calculated at each position in all the copies of the complete genomes of *P. salmonis*. Specific restriction enzymes patterns of *P. salmonis* 16S rDNA for the 13 final selected enzymes are shown. Blue enzymes, enzymes with no cuts in variable regions (green peaks and gray projections).

To identify the genomic variability of *P. salmonis* and to design a PCR-RFLP assay useful to detect all strains known of this species, we retrieved the complete set of 16S rDNA sequences of *P. salmonis*. Thirty six sequences (trimmed and non-redundant), that represent the maximum genomic variability described for this gene, were selected and used (Supplementary Table 1) to construct a phylogenetic tree by maximum-likelihood (Figure 1A). The results showed that the Chilean isolates are separated into two large genogroups (gray and blue branches), which included separately the genogroups A1-15972 (EM-90-like) and B1-32597 (LF-89-like) described recently by Bohle et al. (2014), and placed some non-Chilean isolates in the external clades (IRE-91A, IRE-98-A and Greece). Interestingly, these results suggest that although *P. salmonis* was described for the first time in Chile, its origin could be foreign.

With this information, and to increase the probability to find the highest number of restriction enzymes recognition sites, we selected 15 complete non-redundant 16S rDNA *P. salmonis* sequences that contained conserved regions corresponding to the primers 27F and 1492R (15 bold strains in Supplementary Table 1). From the 4379 enzymes present in REBASE, we selected the 650 restriction enzymes that were commercially available, since this assay is meant to be used in any laboratory. From the 650 enzymes, 152 digest all *P. salmonis* sequences and only 65 recognized conserved restriction sites in the complete set of sequences, generating the same/similar restriction pattern (same number of bands and similar sizes). When comparing the bands patterns generated by these 65 enzymes between *P. salmonis* and the other bacterial sequences present in Greengenes, SILVA and RDP databases, we found that 52 enzymes (bad enzymes) generated identical band patterns among the groups (mistakable) (Supplementary Table 3) and 13 enzymes (good enzymes) produced differential band patterns (distinguishable) (Figure 1B, Supplementary Table 2). Subsequently, the selected 13 restriction enzymes where rigorously characterized and analyzed in order to select the better suited enzyme for the specific PCR-RFLP *P. salmonis* detection and differentiation assay (Supplementary Table 4). Our first analysis was the exploration of the intergenomic and intragenomic variation of the 16S rDNA *P. salmonis* non-redundant sequences, emphasizing in the conserved and variable sites and regions present (Figure 1C). The result shows absence of intragenomic variation among 16S rDNA gene and presence of variable regions among the 16S rDNA sequences (intergenomic variation), noticing for example high variability around 800, 900, and 1000 bp and a large conserved region between 1150 and 1350 bp. This information allowed us to discard the restriction enzymes *Fnu*II, *Asu*I, *Fok*I, *Eco*57I that recognized some restriction sites contained within variable regions, since they are more susceptible of acquiring future nucleotidic variations and with this, the potential generation of different band patterns (gray enzymes in Figure 1C). To simulate experimental conditions, and as a result of the resolution threshold of standard 2% w/v agarose gel electrophoresis, only fragments superior than 90 bp were considered as informative in the analysis. Hence, we discarded the enzymes *Aju*I, *Bse*GI, *Msl*I, and *Oli*I that generated less than four informative bands. Also, we discarded the enzymes that generated fragments with low separation between bands (*Bbv*I, *Hpa*II, and *Mbo*II), and could therefore be difficult to discriminate in agarose gels. Finally, we selected *Pma*CI for our specific PCR-RFLP *P. salmonis* assay, because it achieved the previous requisites (easily discernable), was the cheapest restriction enzyme from the group (Table 1) and had isoschizomers (*Pml*I, *Acv*I, *Bbr*PI, *Eco*72I, and *Psp*CI), relevant aspects for its commercial availability and massive use.

### Validation of specific PCR-RFLP *P. salmonis* assay using *Pma*CI

**Figure 2:**
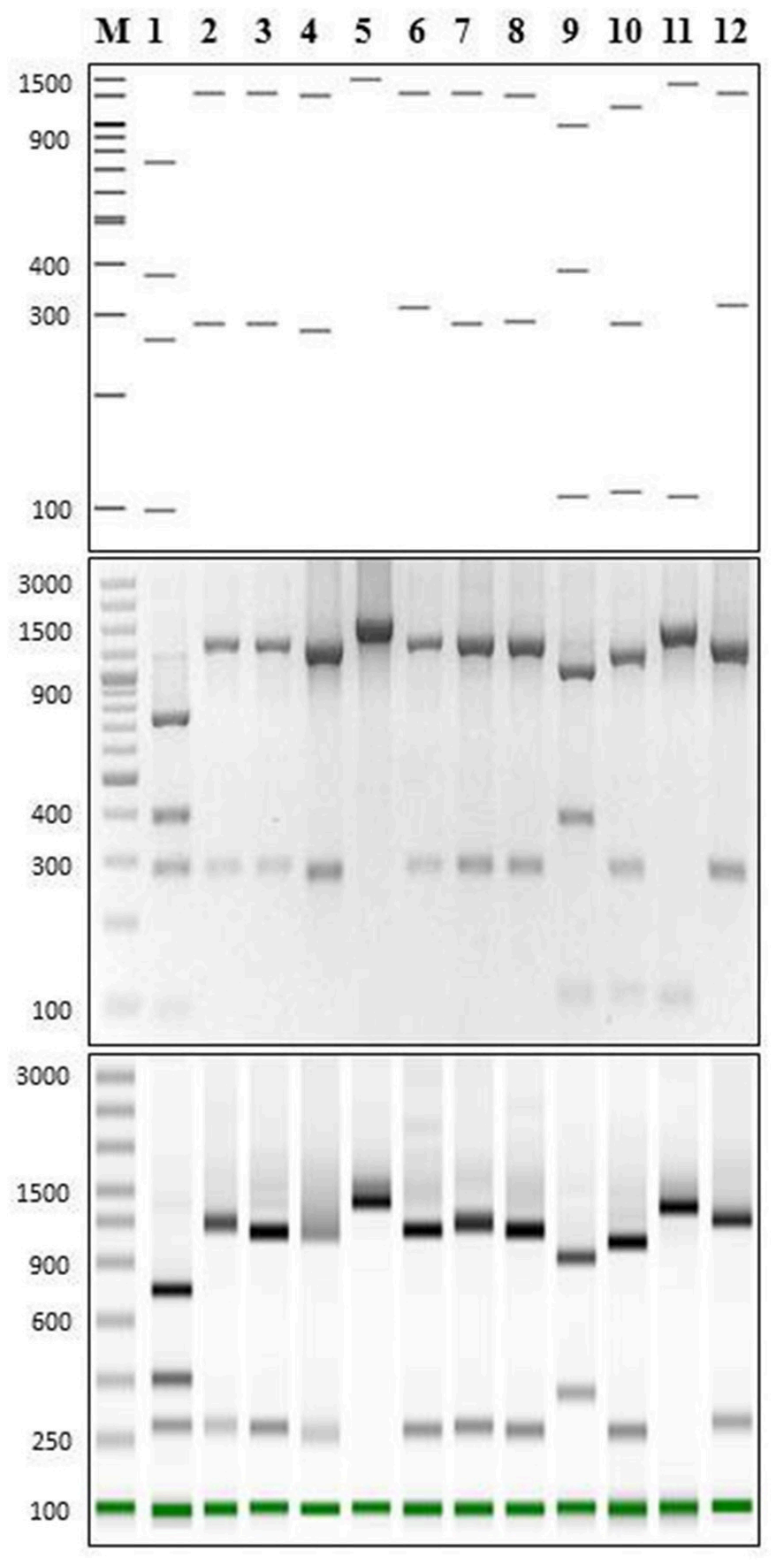
**PCR-RFLP for ***P. salmonis*** and cohabitant strains using PmaCI restriction enzyme. (Upper panel)** Predicted digestion patterns of 16S rDNA using NEBcutter web tool for all bacterial strains used in this study. **(Middle panel)** 2% gel electrophoresis showing PCR-RFLP digestion pattern (16SrDNA amplification using primers 27F/1492R) for all bacterial strains used in this study. **(Lower panel)** Tape Station 2200 screening of PCR-RFLP digestion pattern of samples in B. In all cases: 1, *Piscirickettsia salmonis* LF-89; 2, *Vibrio anguillarum* INTA11; 3, *Aeromonas salmonicida* INTA1; 4, *Flavobacterium psychrophilum* INTA9; 5, *Renibacterium salmoninarum* INTA10; 6, *Shewanella frigidimarina* INTA2; 7, *Photobacterium phosphoreum* INTA3; 8, *Psychrobacter nivimaris* INTA4; 9, *Arthrobacter oxydans* INTA5; 10, *Staphylococcus saprophyricus* INTA6; 11, *Microbacterium lacus* INTA7; 12, *Escherichia coli* INTA8. M, O'GeneRuler 100 bp DNA Ladder Plus (bp).

Using the 15 representatives of *P. salmonis, Pma*CI generated four *in silico* bands of 97.0 ± 0.0; 280.9 ± 0.4; 395.2 ± 1.6 and 731.9 ± 2.1 bp. As this pattern was predicted to be different from the other bacteria, we wanted to experimentally validate this estimation by comparing the pattern of *P. salmonis* with the band pattern of 11 different species that cohabit in fish tissues or in culture media with *P. salmonis* (Table 2). These strains were isolated from infected fish or from culture media where *P. salmonis* is usually grown (laboratory contaminants) (for details, see Materials and Methods). The predicted digestion patterns using *Pma*CI of the 16S rDNA genes from all bacterial strains used in this study, including *P. salmonis*, are shown in Figure 2 (upper panel). The experimental enzymatic digestion with *Pma*CI and the electrophoresis in agarose gel and ScreenTapes (Figure 2, middle and down panels, respectively), showed that real patterns recovered the predicted patterns. It was clearly observed that *P. salmonis* is easily differentiated from all the other bacterial strains, validating our *in silico* predictive method.

We also performed an evaluation of the sensibility (detection limit) of our *Pma*CI PCR-RFLP assay by using different concentrations of digestion products (Supplementary Figure 3). We observed that the detection limit to visualize all the expected bands in agarose gels was of 250 ng, while using the ScreenTapes of TapeStation 2200 technology the detection limit decreased to 0.25 ng.

### Current *P. salmonis* diagnostic methods are not specific

**Figure 3:**
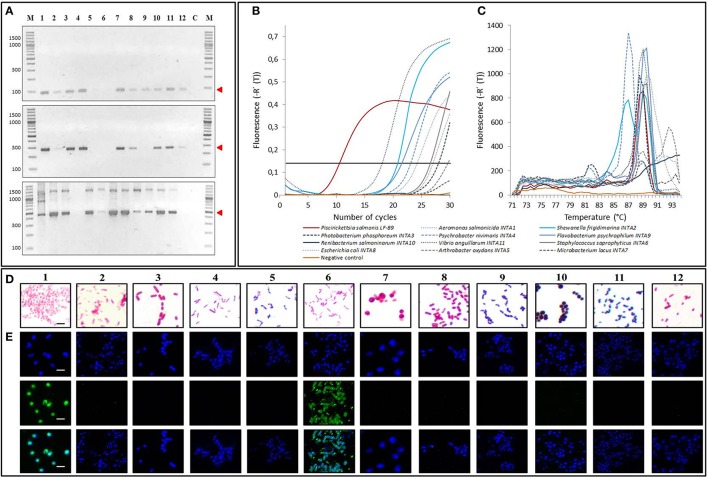
**Specificity of current methods for ***P. salmonis*** diagnosis. (A) (Upper panel)** 2% agarose gel electrophoresis of ITS-PCR assay using primers RTS1/RTS2. Red arrow indicates the 91 bp band amplified from *P. salmonis* LF-89 DNA but also from DNA from most other strains used in this study. **(Middle panel)** 2% agarose gel electrophoresis of ITS-PCR assay using primers RTS1/RTS4. Red arrow indicates the 283 bp band amplified from *P. salmonis* DNA but also from DNA from most other strains used in this study. **(Lower panel)** 2% agarose gel electrophoresis of Nested PCR (second amplification primers PS2S/PS2AS). Red arrow shows the band amplified from *P. salmonis* DNA and from DNA from several of the other strains used in this study (469 bp). M, O'GeneRuler 100 bp DNA Ladder Plus; C, negative amplification control. **(B)** Real time PCR amplification curves using primers Ps16Sreal-F1/Ps16Sreal-R. Threshold: 0.14 Fluorescence [-R' (T)] (black continued line). **(C)** Real time PCR melting curves using primers Ps16Sreal-F1/Ps16Sreal-R. **(D)** Light microscopy images of bacteria after Gram staining. Black bar corresponds to 1.25 5m. **(E)** Confocal microscopy of bacteria stained with DAPI **(Upper panels)**, with a commercial FITC-labeled *P. salmonis* antibody **(Middle panels)** and merged images between DAPI staining and FITC-labeled *P. salmonis* antibody **(Lower panel)**. White bars correspond to 2.5 5m. For **(A,D,E)** 1, *Piscirickettsia salmonis* LF-89; 2, *Vibrio anguillarum* INTA11; 3, *Aeromonas salmonicida* INTA1; 4, *Flavobacterium psychrophilum* INTA9; 5, *Renibacterium salmoninarum* INTA10; 6, *Shewanella frigidimarina* INTA2; 7, *Photobacterium phosphoreum* INTA3; 8, *Psychrobacter nivimaris* INTA4; 9, *Arthrobacter oxydans* INTA5; 10, *Staphylococcus saprophyricus* INTA6; 11, *Microbacterium lacus* INTA7; 12, *Escherichia coli* INTA8.

Six methods are currently used to detect *P. salmonis* in infected fish tissues: ITS-PCR, Nested-PCR, Real time PCR, Taqman probe assay, Gram staining and immunofluorescent detection. We performed all these assays using samples of *P. salmonis* and the 11 strains that cohabit with *P. salmonis*, in order to compare the detection capacity and specificity of our PCR-RFLP assay (Figure 3, Supplementary Table 4). ITS-PCRs consist of two amplification reactions pursued by different sets of primers, being the first amplification performed using primers EubB and EubA, and a seconds amplification using either primer pairs RTS1/RTS2 or RTS1/RTS4 (Marshall et al., 1998) (for details see Materials and Methods). When performing these amplification assays, we could observe the sized bands described for *P. salmonis* identification (RTS1/RTS2: 91 bp and RTS1/RTS4: 283bp), yet most of the other strains also amplified the same sized product (Figure 3A, upper and middle panels). In the case of Nested-PCR, we performed it as described by Corbeil and Crane (2009). As defined, this assay permitted the detection of *P. salmonis*, showing the described sized band of 469 bp together with other unspecific bands (Figure 3A, lower panel). However, the detection was not specific for *P. salmonis*, showing most of the other strains a comparable sized amplification product. We also performed the Real time PCR reaction described as specific for *P. salmonis* using primers 16SRNA-F1 and 16SRNA-R (Karatas et al., 2008). This reaction resulted positive and efficient for *P. salmonis* detection, showing the beginning of the amplification at initial cycles of the reaction (Figures 3B,C, Supplementary Table 4). Nevertheless, this method was not *P. salmonis* specific, resulting also positive (Ct < 30 cycles) for most of the other strains used in this study, except for *Microbacterium lacus* and *Renibacterium salmoninarum*. In the same way, when testing the described *P. salmonis* Taqman probe using primers F-760/R-836 (Corbeil et al., 2003) on all genomic DNA of bacterial isolates, the assay resulted positive (Ct < 30 cycles) for most of the samples (Supplementary Table 4), excluding *Escherichia coli* and *Renibacterium salmoninarum*, implying no real probe specificity for *P. salmonis*. Moreover, our Gram staining results indicated that the differentiation of *P. salmonis* with all the other gram negative strains was not obvious, especially when compared with *Vibrio anguillarum* (Figure 3D). Finally, we prepared immune detection assays using the commercial antibody against *P. salmonis* (GrupoBios) and using DAPI to stain the DNA (Figure 3E). We observed that the antibody efficiently detected *P. salmonis*, but as in all the other assays tested previously, it was not specific, giving also positive signal for *Shewanella frigidimarina*. In summary, all diagnostic methods in current use undeniably detect *P. salmonis*, however none of them is specific.

### PCR-RFLP assay permits discrimination of *P. salmonis* in mixed bacterial cultures

**Figure 4:**
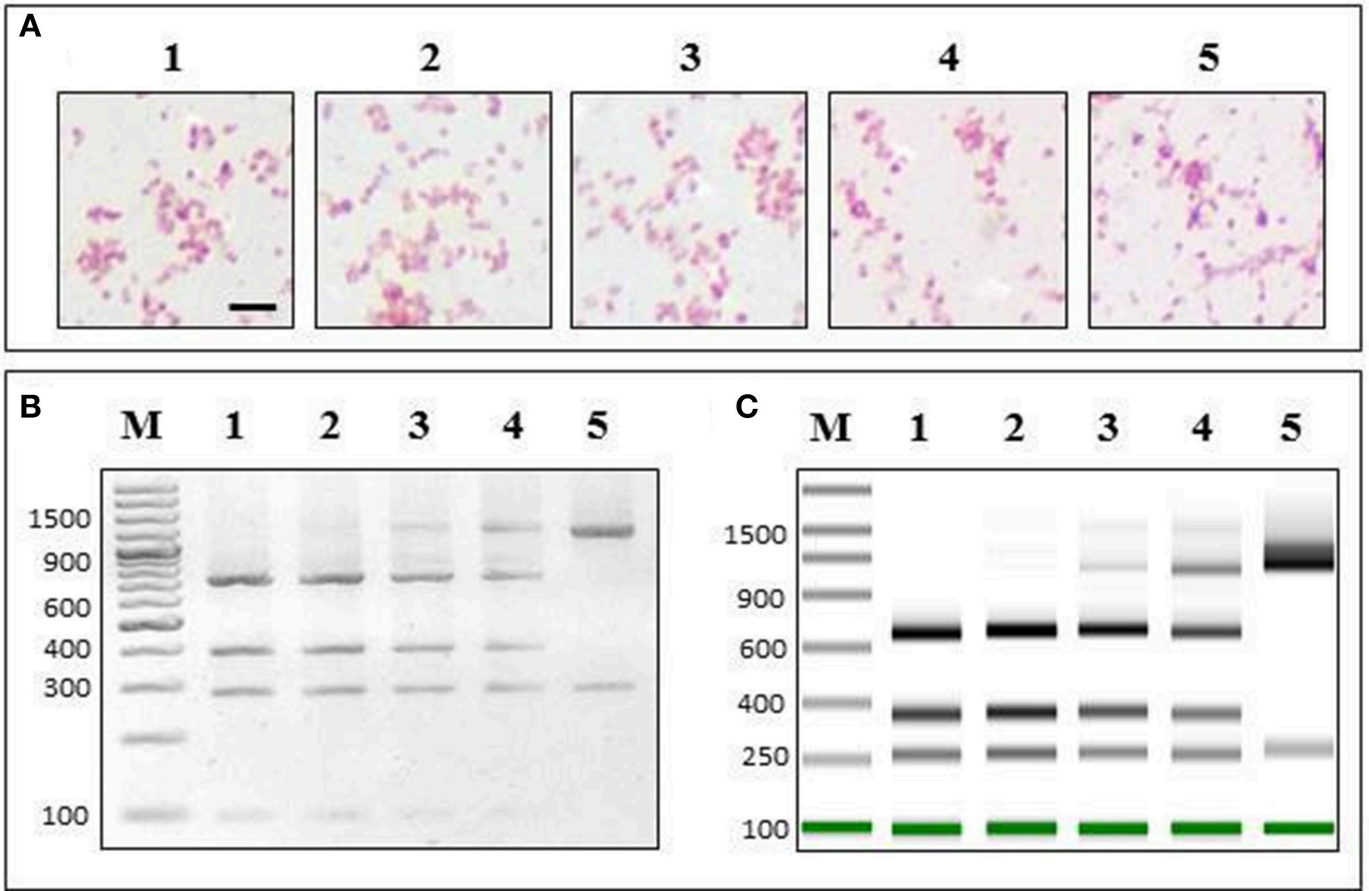
**PCR-RFLP specifically detects ***P. salmonis*** in a mixed sample**. *P. salmonis* LF-89 and *V. anguillarum* INTA11 (in the same growth exponential state) were mixed in sterile culture media at different proportions [*P. salmonis* LF-89 (%)/V. anguillarum INTA11 (%)]: 1, 100/0; 2, 75/25; 3, 50/50; 4, 25/75; 5, 0/100. **(A)** The mixed samples were subjected to Gram staining. Bar corresponds to 1.25 5m. **(B)** 2% agarose gel electrophoresis of PCR-RFLP of the mixed sample using restriction enzyme PmaCI. **(C)** ScreenTape in the Tape Station 2200 instrument of PCR-RFLP of the mixed sample using restriction enzyme PmaCI. M, O'GeneRuler 100 bp DNA Ladder Plus.

In order to test if our PCR-RFLP assay could discriminate *P. salmonis* in a contaminated culture media that also contained a similar gram negative bacterium (*V. anguillarum*), we developed a mixed sample assay (for details see Materials and Methods) (Figure 4). We showed that when mixing *P. salmonis* at different proportions with *V. anguillarum*, the Gram staining was not sensitive enough to separately identify each bacterium (Figure 4A). On the other hand, our PCR-RLFP assay could discriminate them at every mixing proportion, showing how the *P. salmonis* pattern decreased while the *V. anguillarum* pattern signal increased accordingly with the augmented proportion of *V. anguillarum* in the sample (Figure 4B, left). The resulted enzymatic digestion was also analyzed in TapeStation 2200, showing the same different proportion patterns of *P. salmonis* and *V. anguillarum* (Figure 4B, right).

### PCR-RFLP assay permits discrimination of *P. salmonis* in different tissues and fish species

**Figure 5:**
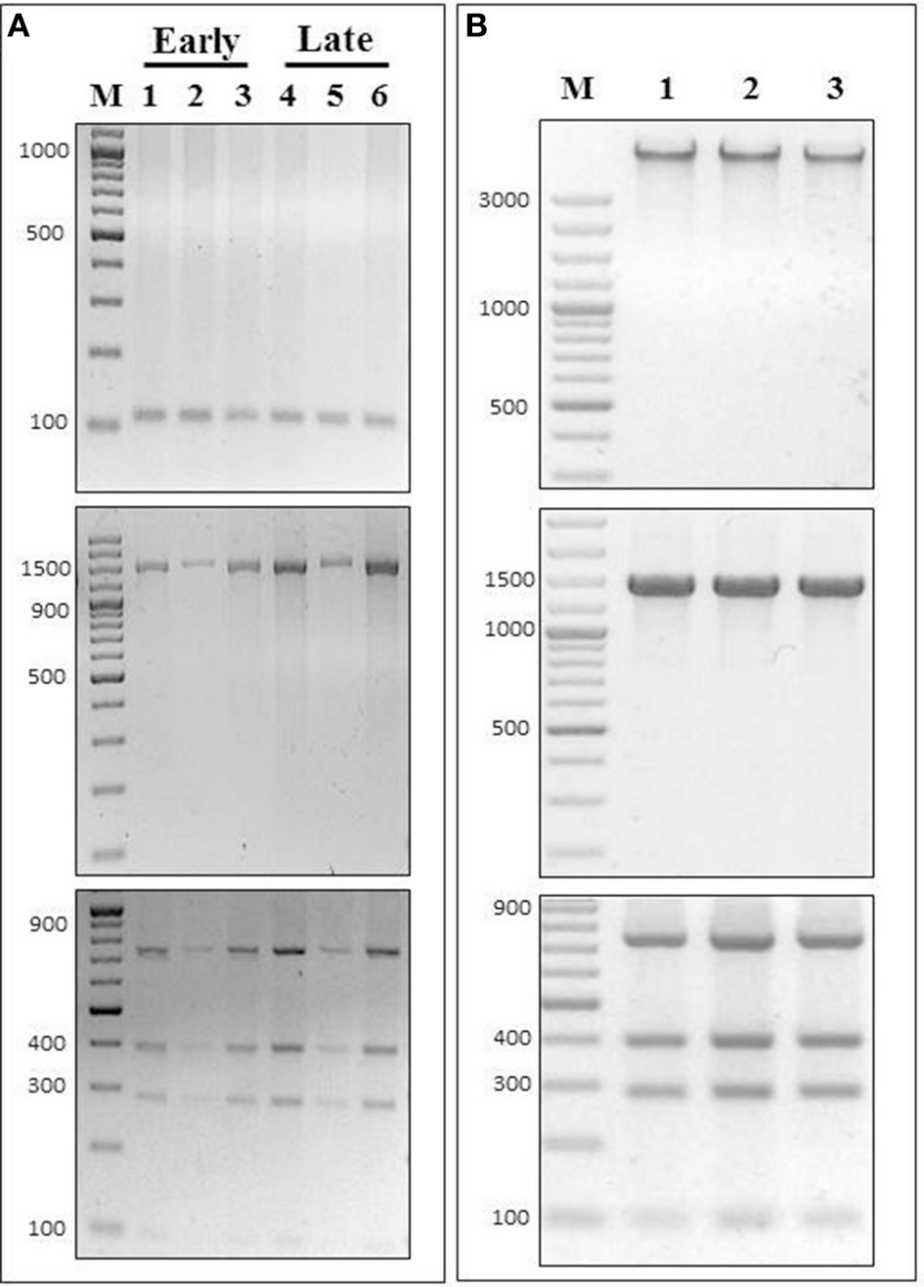
**Detection and identification of ***P. salmonis*** in different fish tissues and species. (A)** DNA extracted from *P. salmonis* LF-89 infected Salmo salar tissues were used to perform PCR-RFLP assays using PmaCI in early or late state of fish infection. **(Upper panel)** PCR amplification of Salmo salar 60S rDNA gene (Ss60sS27F/Ss60s27R), **(Middle panel)** 16S rDNA PCR amplification (using primers 27F/1492R) and **(Lower panel)** PCR-RFLP digestion pattern using enzyme PmaCI. In all cases: 1 and 4, head kidney samples; 2 and 5, spleen samples; 3 and 6, brain samples; M, O'GeneRuler 100 bp DNA Ladder Plus. **(B)** DNA extracted from *P. salmonis* LF-89 infected tissues from 1, *Oncorhynchus tshawytscha* (CHSE-214 embrionic cells); 2, *Oncorhynchus mykiss* (head kidney); 3, *Oncorhynchus kisutch* (head kidney), were used to perform PCR-RFLP assay using restriction enzyme PmaCI. **(Upper panel)** Genomic DNA visualized in a 2% agarose gel electrophoresis, **(Middle panel)** 16S rDNA amplicon (using primers 27F/1492R) visualized in a 2% agarose gel electrophoresis, and **(Lower panel)** PCR-RFLP digestion pattern visualized in a 2% agarose gel electrophoresis. M, O'GeneRuler 100 bp DNA Ladder Plus.

To test if our PCR-RFLP assay could discriminate *P. salmonis* in different infected tissues, we applied our method in DNA extracted from head kidney, spleen and brain samples of Atlantic salmon at different *P. salmonis* infection states. These are complex samples, where the proportion of *P. salmonis* is lower than in a culture media and DNA from the fish is also present in the DNA extractions. In order to test DNA extractions from the infected fish tissues, a specific salmon PCR was performed to each sample. An amplicon of expected size was observed in every case, confirming the presence of salmon DNA in the samples (Figure 5A, upper panel). Further, these same samples were used to amplify the 16S rDNA gene using universal bacterial primers (27F/1492R). The amplicons of the expected sizes were visualized in an agarose gel electrophoresis (Figure 5A, middle panel). In order to make sure that the infection corresponded exclusively to *P. salmonis*, we sequenced these amplicons, which all corresponded to the 16S rDNA sequence of *P. salmonis.* Thus, we proved that the tissues used to test our PCR-RFLP assay were only contaminated with this bacterium. Then, each 16S rDNA amplicon was digested using the enzyme *Pma*CI, where the predicted restriction pattern for *P. salmonis* was observed every time (Figure 5A, lower panel). Hence, our PCR-RFLP method was validated in these complex samples. We analyzed an “early” stage of infection, which is determined as the period of fish survival when mortalities produced by *P. salmonis* infection are not significant, and also we analyzed the time of peak of mortalities, established as the “late” stage of infection (for details, see Material and Methods). At both stages, and in all the infected tissues tested, our PCR-RFLP assay detected *P. salmonis*. Healthy fish were used as experimental controls, where no amplification of the 16S rDNA gene was observed.

We also wanted to test if our PCR-RFLP assay could detect *P. salmonis* in infected tissues of different fish species. For this reason, we applied our method in DNA extracted from infected *Oncorhynchus tshawytscha* CHSE-214 embryonic cells, *Oncorhynchus mykiss* head kidney and *Oncorhynchus kisutch* head kidney. In all three samples, the DNA extracted were of high integrity (Figure 5B, upper panel), and were used as templates for PCR amplifications using the 16S rDNA bacterial universal primers previously mentioned (Figure 5B, middle panel). The amplicons were sequenced and all corresponded exclusively to *P. salmonis*, proving that the cells and tissues were only contaminated with this bacterium. Then, and in order to test our PCR-RFLP method in these different fish species, each 16S rDNA amplicon was digested using the enzyme *Pma*CI, where the *P. salmonis* restriction pattern was confirmed in every sample (Figure 5B, lower panel). Non-infected cells and healthy fish were used as experimental controls, where no amplification of the 16S rDNA gene was observed.

## Discussion

In this study, by computer-simulation analyses we identified a group of restriction enzymes that generated unique *P. salmonis* 16S rDNA band patterns, distinguishable from all other bacteria. Then, we designed and experimentally validated a PCR-RFLP assay using *Pma*CI restriction enzyme by comparing the band patterns of *P. salmonis* with those generated by bacteria that cohabit with this bacterium (fish bacterial isolates and culture media laboratory contaminants). *Piscirickettsia salmonis* was chosen as the target microorganism because it is an important fish pathogen, it is the single species in its genus and the six genomics copies of its 16S rDNA gene are identical (intragenomic conservation) (Pulgar et al., 2015b). This features make *P. salmonis* an ideal candidate to try a PCR-RFLP detection assay, yet the described pipeline can be used to identify restriction enzymes that generate band patterns specific for any bacterial species.

PCR-RFLP has been described as an appropriate assay for microbial diversity characterization (Moyer et al., 1994; Haddad et al., 1995) and an efficient species/strain-specific detection and differentiation tool (bacterial genotyping) (Jayarao et al., 1991; Vaneechoutte et al., 1992, 1993; Pleckaityte et al., 2012; Cheraghchi et al., 2014; Öztürk and Meterelliyöz, 2015). However, one of the weaknesses of designing and developing a PCR-RFLP method is to determine how many and which genes and restriction enzymes must be used for an efficient analysis.

In this study, we selected the 16S rDNA gene (SSU, small subunit of ribosomal RNA) to develop our PCR-RFLP assay, because of its genetic characteristics, like that it is universal (present in all bacteria) and its possession of conserved and variable regions (Woese and Fox, 1977), and massive sequence information available, which is deposited in public specialized and cured databases (Greengenes, SILVA and RDP databases). These features facilitate the use of this gene to design molecular tools, as it allows access to the information of the species of interest as well as the information related to all other species, essential aspect for the specific detection of environmental microorganisms. In this sense, it is important to mention that even though we selected all full-length 16S rDNA gene sequences and then trimmed them with the universal primers, the general taxa composition was maintained besides the significant decrease of sequence representation after trimming (Supplementary Figure 2), thus conserving the taxonomic diversity. Taken all these aspects in consideration, the unspecificity observed in the current methods to detect *P. salmonis* could be partly explained by the limited microbiological and genomic information available when the primers, probes and antibodies were designed.

Regarding the selection of restriction enzymes for PCR-RFLP assays, most studies report that their selection is arbitrary, but usually are tetra or hexacutter, which is based in the random frequency of restriction sites recognition and the number of visible bands generated in a sequence of about 1500 bp (like the 16S rDNA gene). However, this approach does not consider the position of the restriction sites recognized by the enzymes, which can be found in regions of high variability and may generate different band patterns among strains of the interested species. In addition, most of these assays need to use more than one enzyme to overcome the probability of generating mistakable band patterns with closely related bacteria, but the enzymes not always can be combined in a single reaction. Moreover, to use more than one enzyme separately implicate a more time-consuming, labor-intensive and costly method.

Our genomic-based approach allows to increase the probability of finding a single (or more) enzyme that can distinguish among bacteria, because our inclusion criteria comprises the selection of enzymes that recognize conserved restriction sites in conserved regions of the 16S rDNA gene of the interested species (Figure 1C). In addition, our approach includes the comparison of all band patterns generated by the complete set of commercial enzymes that digest the species of interest and the complete collection of 16S rDNA gene sequences. In our case, we compared the band patterns generated by all commercial restriction enzymes that digest the 15 non-redundant *P. salmonis* sequences (*n* = 152) with the band patterns generated by this restriction enzymes in the other bacteria (*n* = 568, 169), representing a total of 86,361,688 restriction patterns. With this approximation, we were able to choose thirteen enzymes (Figure 1B, Supplementary Table 2) that could distinguish a specific pattern for *P. salmonis* when compared to all other bacteria.

To evaluate experimentally the specificity of our *P. salmonis* PCR-RFLP assay (validation), we developed a concept-test that compared the band patterns of *P. salmonis* with other 11 related bacteria using the restriction enzyme *Pma*CI. This enzyme generates four informative and easily recognizable bands in the 16S rDNA gene of *P. salmonis*. Being an hexacutter restriction enzyme, in the other bacteria it produced a low number of bands, making them easy to visualize and compare (Figure 2), in contrast to other tested enzymes (data not shown). The experimental approach using *Pma*CI confirmed that the *in silico* pattern produced in *P. salmonis* was distinguishable to the pattern generated in co-habitant bacteria, validating our prediction (Figure 2). Additionally, since the technique involves an enzymatic amplification of the 16S rDNA gene, its detection limit is comparable with all other PCR-based methods used to detect *P. salmonis* in agarose gels, whereas its visualization is widely improved (0.25 ng, 1000-fold improved) using the ScreenTapes of TapeStation 2200 technology, as it has been reported recently (Soler-García et al., 2014). Moreover, the development of new ScreenTapes (high sensitivity D5000 of Agilent Technologies) promises to further improve this detection limit to 0.01 ng, which will give remarkable sensibility to all PCR based methods, including our PCR-RFLP assay.

Furthermore, since our PCR-RFLP was designed to amplify the 16S rDNA gene of eubacteria, we designed and validated a PCR-RFLP assay that not only accurately detects the presence of *P. salmonis* in infected fish tissues or in culture media like other currently used methodologies, but that also has the capacity to detect other bacteria in co-infected fish or contaminated media, simultaneously in the same assay (Figure 4).

The capacity of our specific PCR-RFLP assay to detect low concentrations of bacterial 16S rDNA in different tissues of farmed salmonid species (Figure 5) is a relevant aspect especially in early states of infection, where the signology is absent or unspecific. In this context, the PCR-RFLP assay is a useful tool because it identifies low concentrations of *P. salmonis*, is rapid (lasts around 4 h with FastDigest restriction enzymes), is reproducible (it is routinely used in our laboratory (*n*~1000) to verify the purity of bacterial cultures and the presence of the bacterium in fish tissues) and cheap (it allows the identification of large numbers of samples in any laboratory equipped with a conventional thermo cycler, electrophoresis chamber and basic molecular biology reagents). Thus, the use of our specific *Pma*CI PCR-RFLP assay provides an accessible alternative method to detect and differentiate *P. salmonis* from other bacteria, which represents a fundamental aspect for research purposes, vaccine development and clinical diagnostic of SRS.

## Author contributions

DM and BG carried out the experimental assays, literature search, data analysis, participated in the design of the study and drafted the manuscript, and contributed equally to this work. PA carried out microbiological cultures and some molecular assays. JM carried out the bioinformatic analyses and participated in its discussion. MG and VC participated in its coordination, and have critically examined and corrected the manuscript. RP conceived the study, participated in its design and coordination, and critically examined and corrected the manuscript. All authors read and approved the final manuscript.

### Conflict of interest statement

The authors declare that the research was conducted in the absence of any commercial or financial relationships that could be construed as a potential conflict of interest.

## Footnotes

^1^www.sernapesca.cl https://www.sernapesca.cl/index.php?option=com_remository&Itemid=246&func=select&id=419, www.sernapesca.cl https://www.sernapesca.cl/index.php?option=com_remository&Itemid=246&func=startdown&id=6726.
