# Supplementary material for: Genomic-Based Restriction Enzyme Selection for Specific Detection of Piscirickettsia salmonis by 16S rDNA PCR-RFLP
Source: Front Microbiol. 2016 May 9;7:643. doi: 10.3389/fmicb.2016.00643 (PMC4860512; doi:10.3389/fmicb.2016.00643)
Supplement: Supplementary file 2 [file Table2.DOCX]

| **650 REBASE commercial enzymes** |
| --- |
| *AanI, AccB7I, Acc65I, AatII, AbaSI, AarI, AccI, AbsI, AccBSI, AccB1I, Acc16I, AasI, Acc36I, AccII, AccIII, AciI, AclI, AclWI, AcoI, AcsI, AcuI, AcvI, AcyI, AdeI, AfaI, AfeI, AfiI, AflII, AflIII, AgeI, AgsI, AhdI, AhlI, AjiI, AjnI, AjuI, AleI, AloI, AluBI, AluI, Alw21I, Alw26I, Alw44I, AlwI, AlwNI, Ama87I, Aor13HI, Aor51HI, ApaI, ApaLI, ApeKI, ApoI, ArsI, AscI, AseI, AsiGI, AsiSI, Asp700I, Asp718I, AspA2I, AspLEI, AspS9I, AsuC2I, AsuHPI, AsuI, AsuII, AsuNHI, AvaI, AvaII, AvrII, AxyI, BaeGI, BaeI, BalI, BamHI, BanI, BanII, BarI, BauI, BbrPI, BbsI, Bbv12I, BbvCI, BbvI, BccI, BceAI, BcgI, BciT130I, BciVI, BclI, BcnI, BcoDI, BcuI, BfaI, BfmI, BfoI, BfrI, BfuAI, BfuCI, BfuI, BglI, BglII, BisI, BlnI, BlpI, BlsI, BmcAI, Bme1390I, Bme18I, BmeRI, BmeT110I, BmgBI, BmgT120I, BmiI, BmrFI, BmrI, BmsI, BmtI, BmuI, BoxI, BpiI, BplI, BpmI, Bpu10I, Bpu1102I, Bpu14I, BpuEI, BpuMI, Bsa29I, BsaAI, BsaBI, BsaHI, BsaI, BsaJI, BsaWI, BsaXI, Bsc4I, Bse118I, Bse1I, Bse21I, Bse3DI, Bse8I, BseAI, BseBI, BseCI, BseDI, BseGI, BseJI, BseLI, BseMI, BseMII, BseNI, BsePI, BseRI, BseSI, BseX3I, BseXI, BseYI, BsgI, Bsh1236I, Bsh1285I, BshFI, BshNI, BshTI, BshVI, BsiEI, BsiHKAI, BsiHKCI, BsiSI, BsiWI, BslFI, BslI, BsmAI, BsmBI, BsmFI, BsmI, BsnI, Bso31I, BsoBI, Bsp119I, Bsp120I, Bsp1286I, Bsp13I, Bsp1407I, Bsp143I, Bsp1720I, Bsp19I, Bsp68I, BspACI, BspANI, BspCNI, BspDI, BspEI, BspFNI, BspHI, BspLI, BspMAI, BspMI, BspOI, BspPI, BspQI, BspT104I, BspT107I, BspTI, BspTNI, BsrBI, BsrDI, BsrFI, BsrGI, BsrI, BsrSI, BssAI, BssECI, BssHII, BssMI, BssNAI, BssNI, BssSI, BssT1I, Bst1107I, Bst2BI, Bst2UI, Bst4CI, Bst6I, BstACI, BstAFI, BstAPI, BstAUI, BstBAI, BstBI, BstC8I, BstDEI, BstDSI, BstEII, BstENI, BstF5I, BstFNI, BstH2I, BstHHI, BstKTI, BstMAI, BstMBI, BstMCI, BstMWI, BstNI, BstNSI, BstOI, BstPAI, BstPI, BstSCI, BstSFI, BstSLI, BstSNI, BstUI, BstV1I, BstV2I, BstX2I, BstXI, BstYI, BstZ17I, BstZI, Bsu15I, Bsu36I, BsuI, BsuRI, BsuTUI, BtgI, BtgZI, BtrI, BtsCI, BtsI, BtsIMutI, BtuMI, BveI, Cac8I, CaiI, CciI, CciNI, CfoI, Cfr10I, Cfr13I, Cfr42I, Cfr9I, ClaI, CpoI, CseI, CsiI, Csp6I, CspAI, CspCI, CspI, CviAII, CviJI, CviKI-1, CviQI, DdeI, DinI, DpnI, DpnII, DraI, DraIII, DrdI, DriI, DseDI, EaeI, EagI, Eam1104I, Eam1105I, EarI, EciI, Ecl136II, EclXI, Eco105I, Eco130I, Eco147I, Eco24I, Eco31I, Eco32I, Eco47I, Eco47III, Eco52I, Eco53kI, Eco57I, Eco72I, Eco81I, Eco88I, Eco91I, EcoICRI, EcoNI, EcoO109I, EcoO65I, EcoP15I, EcoRI, EcoRII, EcoRV, EcoT14I, EcoT22I, EcoT38I, EgeI, EheI, ErhI, Esp3I, FaeI, FaiI, FalI, FaqI, FatI, FauI, FauNDI, FbaI, FblI, Fnu4HI, FnuDII, FokI, FriOI, FseI, Fsp4HI, FspAI, FspBI, FspEI, FspI, GlaI, GluI, GsaI, GsuI, HaeII, HaeIII, HapII, HgaI, HhaI, Hin1I, Hin1II, Hin6I, HincII, HindII, HindIII, HinfI, HinP1I, HpaI, HpaII, HphI, Hpy166II, Hpy188I, Hpy188III, Hpy8I, Hpy99I, HpyAV, HpyCH4III, HpyCH4IV, HpyCH4V, HpyF10VI, HpyF3I, HpySE526I, Hsp92I, Hsp92II, HspAI, KasI, KflI, Kpn2I, KpnI, KroI, Ksp22I, KspAI, KspI, Kzo9I, LguI, LmnI, LpnPI, Lsp1109I, LweI, MabI, MaeI, MaeII, MaeIII, MalI, MauBI, MbiI, MboI, MboII, MfeI, MflI, MhlI, MlsI, MluCI, MluI, MluNI, Mly113I, MlyI, MmeI, MnlI, Mox20I, Mph1103I, MreI, MroI, MroNI, MroXI, MscI, MseI, MslI, Msp20I, MspA1I, MspCI, MspI, MspJI, MspR9I, MssI, MunI, Mva1269I, MvaI, MvnI, MwoI, NaeI, NarI, NciI, NcoI, NdeI, NdeII, NgoMIV, NheI, NlaIII, NlaIV, NmeAIII, NmuCI, NotI, NruI, NsbI, NsiI, NspI, NspV, OliI, PacI, PaeI, PaeR7I, PagI, PalAI, PasI, PauI, PceI, PciI, PciSI, PcsI, PctI, PdiI, PdmI, PfeI, Pfl23II, PflFI, PflMI, PfoI, PinAI, Ple19I, PleI, PluTI, PmaCI, PmeI, PmlI, PpsI, Ppu21I, PpuMI, PscI, PshAI, PshBI, PsiI, Psp124BI, Psp1406I, Psp5II, Psp6I, PspCI, PspEI, PspFI, PspGI, PspLI, PspN4I, PspOMI, PspPI, PspPPI, PspXI, PsrI, PstI, PstNI, PsuI, PsyI, PteI, PvuI, PvuII, RgaI, RigI, RruI, RsaI, RsaNI, RseI, Rsr2I, RsrII, SacI, SacII, SalI, SapI, SaqAI, SatI, Sau3AI, Sau96I, SauI, SbfI, ScaI, SchI, ScrFI, SdaI, SduI, SetI, SexAI, SfaAI, SfaNI, SfcI, SfiI, SfoI, Sfr274I, Sfr303I, SfuI, SgeI, SgfI, SgrAI, SgrBI, SgrDI, SgsI, SinI, SlaI, SmaI, SmiI, SmiMI, SmlI, SmoI, SnaBI, SpeI, SphI, SrfI, Sse8387I, Sse9I, SseBI, SsiI, SspDI, SspI, SspMI, SstI, StuI, StyD4I, StyI, SwaI, TaaI, TaiI, TaqI, TaqII, TasI, TatI, TauI, TfiI, Tru1I, Tru9I, TscAI, TseFI, TseI, Tsp45I, TspDTI, TspGWI, TspMI, TspRI, Tth111I, Van91I, Vha464I, VneI, VpaK11BI, VspI, XagI, XapI, XbaI, XceI, XcmI, XhoI, XmaI, XmaJI, XmiI, XmnI, XspI, ZraI, ZrmI, Zsp2I, Bce83I, BetI, BfiI, BinI, BsiI, BsiYI, BspLU11I, BspMII, BssKI, CauII, CfrI, CviRI, DraII, DsaI, EspI, HgiJII, Hin4II, Hpy178III, McrI, NspBII, SecI, SfeI, Tsp4CI, XhoII, XmaIII* |
| **152 REBASE commercial enzymes that cut all *P. salmonis* non-redundant sequences** |
| *AatII, BbvI, ApoI, AgsI, AjuI, AciI, BcgI, AluI, BceAI, AvaI, AlwNI, AflIII, ApaI, BglII, BsaAI, BseRI, BslFI, BsmAI, BspCNI, BspHI, BspMI, BsrI, BsrDI, BstEII, AcyI, AsuI, BalI, Bce83I, BciT130I, BetI, BfiI, BinI, BisI, BlsI, BseGI, BseMII, BseSI, BsiI, BsiYI, Bsp120I, Bsp1407I, BspMII, BssKI, BstKTI, BtgZI, BtsI, BtsIMutI, Cac8I, CauII, Cfr10I, Cfr9I, CfrI, Csp6I, CviAII, CviJI, CviRI, DdeI, DpnI, DraII, DraIII, DsaI, Eam1105I, EciI, Eco57I, EcoNI, EcoP15I, EcoRII, EcoRV, FaiI, FalI, FatI, FauI, FnuDII, FokI, GlaI, HaeII, HaeIII, HgaI, HgiJII, HhaI, Hin4II, Hin6I, HindII, HinfI, HpaII, HphI, Hpy166II, Hpy178III, Hpy188I, Hpy99I, LpnPI, MaeI, MaeII, MaeIII, MboI, MboII, MluCI, MluI, MlyI, MmeI, MnlI, MseI, MslI, MspJI, MwoI, NcoI, NlaIII, NlaIV, NruI, NspBII, NspI, OliI, PasI, PcsI, PflMI, PleI, PmaCI, PstI, RsaI, SacII, SauI, ScrFI, SduI, SecI, SetI, SexAI, SfaNI, SfeI, SgeI, SmaI, SmlI, SphI, SspI, StuI, StyI, TaiI, TaqI, TatI, TauI, TfiI, TseI, Tsp45I, Tsp4CI, TspDTI, TspGWI, TspRI, Tth111I, VspI, XbaI, XhoII, XmnI, ZraI* |
| **65 REBASE commercial enzymes that generate the same digestion pattern in all non-redundant *P. salmonis* sequences** |
| *AatII, BbvI, AjuI, BcgI, AvaI, AlwNI, BglII, BsaAI, BslFI, BsmAI, BspHI, BspMI, BsrI, BsrDI, BstEII, AcyI, AsuI, BalI, BfiI, BseGI, BseSI, BsiI, Bsp120I, Bsp1407I, BspMII, BtsI, Cfr9I, Eam1105I, EciI, Eco57I, EcoNI, EcoP15I, EcoRV, FalI, FnuDII, FokI, HaeII, HgaI, HpaII, Hpy99I, MboII, MluI, MlyI, MslI, NcoI, NruI, NspBII, OliI, PleI, PmaCI, SacII, SauI, SmaI, SphI, SspI, StuI, TatI, TseI, TspDTI, TspGWI, Tth111I, VspI, XbaI, XmnI, ZraI* |
| **13 REBASE commercial enzymes that generate different digestion patterns in all *P. salmonis* non-redundant sequences compared to other bacteria** |
| *AjuI, AsuI, BbvI, BsaAI, BseGI, Eco57I, FnuDII, FokI, HpaII, MboII, MslI, OliI, PmaCI* |
