# Supplementary material for: Genomic-Based Restriction Enzyme Selection for Specific Detection of Piscirickettsia salmonis by 16S rDNA PCR-RFLP
Source: Front Microbiol. 2016 May 9;7:643. doi: 10.3389/fmicb.2016.00643 (PMC4860512; doi:10.3389/fmicb.2016.00643)
Supplement: Supplementary file 5 [file DataSheet1.DOCX]

Supplementary Material

Genomic-based restriction enzyme selection for specific detection of *Piscirickettsia salmonis* by 16S rDNA PCR-RFLP

Dinka Mandakovic^1, 3, #^, Benjamín Glasner^1, #^, Jonathan Maldonado^1, 3^, Pamela Aravena^1, 2^, Mauricio González^1, 2, 3^, Verónica Cambiazo^1, 2, 3^, Rodrigo Pulgar^1, 2, 3, *^

^1^Laboratorio de Bioinformática y Expresión Génica, INTA-Universidad de Chile, El Líbano 5524, Santiago, Chile.

^2^ Laboratorio de Genómica Aplicada, INTA-Universidad de Chile, El Líbano 5524, Santiago, Chile.

^3^Fondap Center for Genoma Regulation (CGR), Avenida Blanco Encalada 2085, Santiago, Chile.

*Corresponding author: Rodrigo Pulgar, Laboratorio de Bioinformática y Expresión Génica, INTA-Universidad de Chile, El Líbano 5524, Santiago, Chile.

rpulgar@inta.uchile.cl

#These authors have contributed equally to this work

**
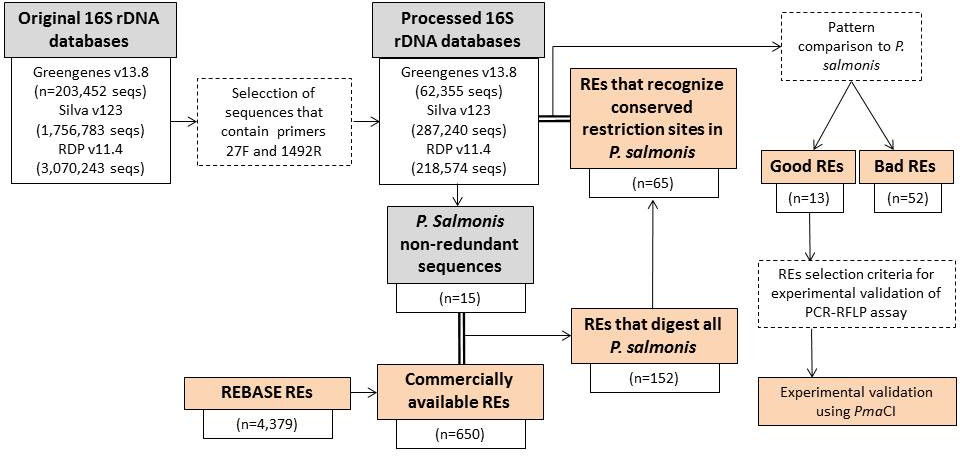
**

**Supplementary Figure 1. Bioinformatic design for selection of enzyme for *P. salmonis* PCR-RFLP specific detection assay.** Seqs: Sequences (in grey); REs: Restriction enzymes (in orange). Good REs: REs that when comparing the “Processed 16S rDNA databases” with *P. salmonis* seqs, produce differentiable patterns. Bad REs: REs that when comparing the Processed 16S rDNA database with *P. salmonis* seqs, do not produce differentiable patterns.


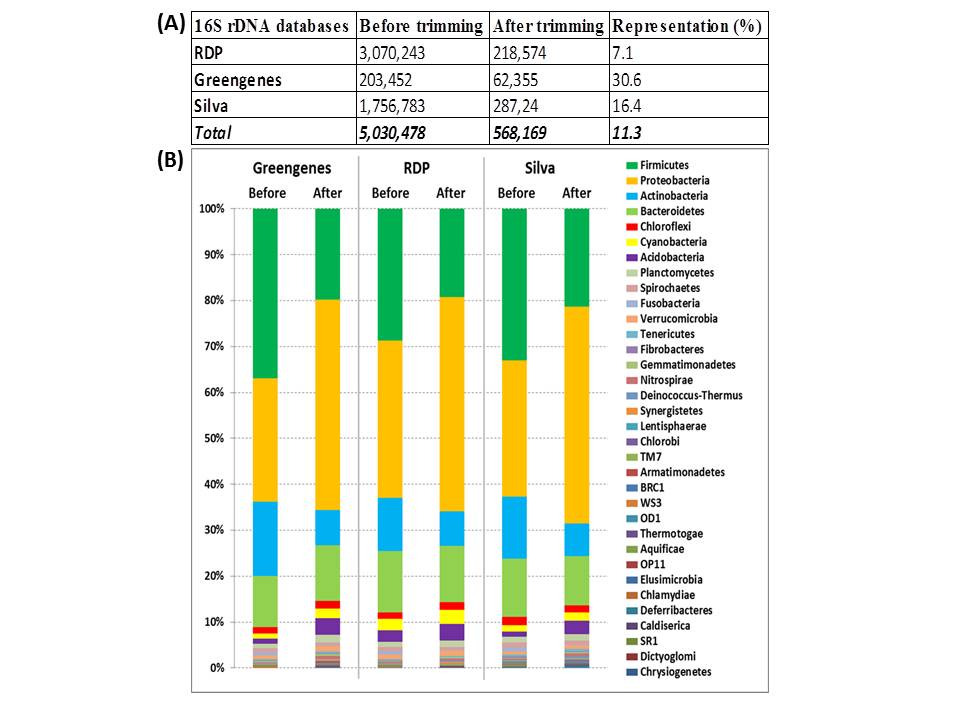


**Supplementary Figure 2.** **Number of sequences and** t**axa composition of databases used in this study before and after trimming the sequences with universal 16S rRNA primers.** (A) Number of sequences before and after trimming. (B) Relative abundance of phyla before and after trimming. Different colors represent different bacterial phyla. Databases used in this study were Greengenes v13.8, RDP v11.4 and Silva v123 databases.

**
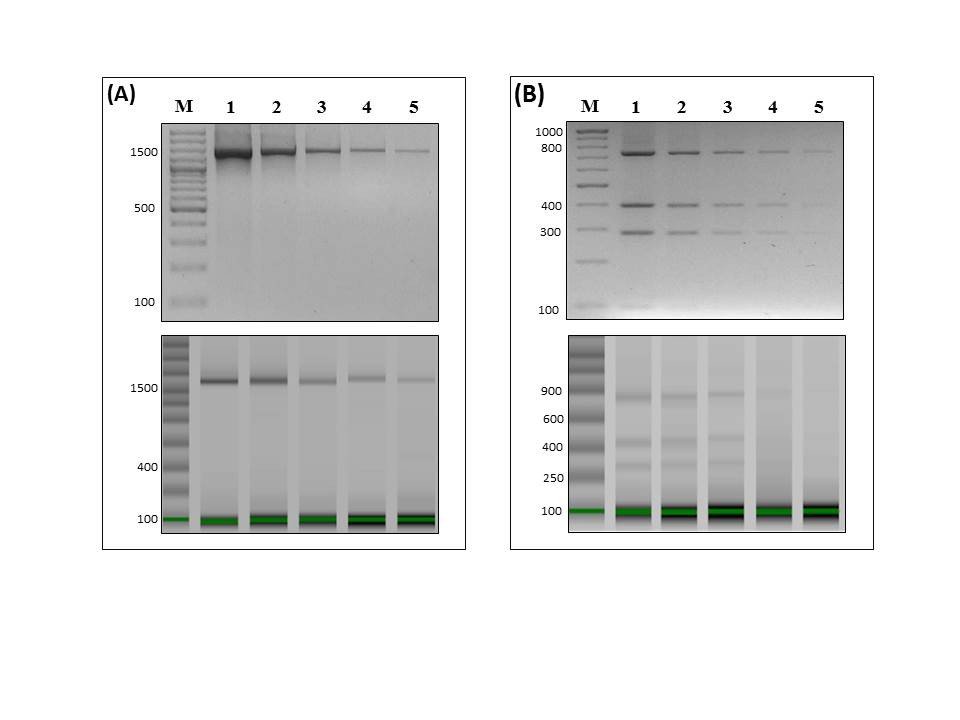
**

**Supplementary Figure 3.** **Sensibility assay.** (A) *P. salmonis* LF-89 16S rRNA amplicons visualized in (Up) 2% agarose gel electrophoresis, M (bp): O´GeneRuler 100 bp DNA Ladder Plus, and visualized in (Down) TapeStation Agilent Technologies. 1: 500 ng PCR product; 2: 250 ng PCR product; 3: 125 ng PCR product; 4: 62.5 ng PCR product; 5: 31.25 ng PCR product. (B) *P. salmonis* LF-89 PCR-RFLP using enzyme *Pma*CI visualized in (Up) 2% agarose gel electrophoresis, M (bp): O´GeneRuler 100 bp DNA Ladder Plus, and visualized in (Down) TapeStation Agilent Technologies. 1: 1 ng PCR product; 2: 0.5 ng PCR product; 3: 0.25 ng PCR product; 4: 0.125 ng PCR product; 5: 0.0625 ng PCR product.

## Supplementary Tables

**Supplementary Table 1. Non-redundant *P. salmonis* 16S rRNA sequences used for Neighborg-joining tree and for restriction enzymes selection.** Bold strains were used for restriction enzymes selection.

**Supplementary Table 2. Restriction enzymes and selection criteria.**

| **650 REBASE commercial enzymes** |
| --- |
| *AanI, AccB7I, Acc65I, AatII, AbaSI, AarI, AccI, AbsI, AccBSI, AccB1I, Acc16I, AasI, Acc36I, AccII, AccIII, AciI, AclI, AclWI, AcoI, AcsI, AcuI, AcvI, AcyI, AdeI, AfaI, AfeI, AfiI, AflII, AflIII, AgeI, AgsI, AhdI, AhlI, AjiI, AjnI, AjuI, AleI, AloI, AluBI, AluI, Alw21I, Alw26I, Alw44I, AlwI, AlwNI, Ama87I, Aor13HI, Aor51HI, ApaI, ApaLI, ApeKI, ApoI, ArsI, AscI, AseI, AsiGI, AsiSI, Asp700I, Asp718I, AspA2I, AspLEI, AspS9I, AsuC2I, AsuHPI, AsuI, AsuII, AsuNHI, AvaI, AvaII, AvrII, AxyI, BaeGI, BaeI, BalI, BamHI, BanI, BanII, BarI, BauI, BbrPI, BbsI, Bbv12I, BbvCI, BbvI, BccI, BceAI, BcgI, BciT130I, BciVI, BclI, BcnI, BcoDI, BcuI, BfaI, BfmI, BfoI, BfrI, BfuAI, BfuCI, BfuI, BglI, BglII, BisI, BlnI, BlpI, BlsI, BmcAI, Bme1390I, Bme18I, BmeRI, BmeT110I, BmgBI, BmgT120I, BmiI, BmrFI, BmrI, BmsI, BmtI, BmuI, BoxI, BpiI, BplI, BpmI, Bpu10I, Bpu1102I, Bpu14I, BpuEI, BpuMI, Bsa29I, BsaAI, BsaBI, BsaHI, BsaI, BsaJI, BsaWI, BsaXI, Bsc4I, Bse118I, Bse1I, Bse21I, Bse3DI, Bse8I, BseAI, BseBI, BseCI, BseDI, BseGI, BseJI, BseLI, BseMI, BseMII, BseNI, BsePI, BseRI, BseSI, BseX3I, BseXI, BseYI, BsgI, Bsh1236I, Bsh1285I, BshFI, BshNI, BshTI, BshVI, BsiEI, BsiHKAI, BsiHKCI, BsiSI, BsiWI, BslFI, BslI, BsmAI, BsmBI, BsmFI, BsmI, BsnI, Bso31I, BsoBI, Bsp119I, Bsp120I, Bsp1286I, Bsp13I, Bsp1407I, Bsp143I, Bsp1720I, Bsp19I, Bsp68I, BspACI, BspANI, BspCNI, BspDI, BspEI, BspFNI, BspHI, BspLI, BspMAI, BspMI, BspOI, BspPI, BspQI, BspT104I, BspT107I, BspTI, BspTNI, BsrBI, BsrDI, BsrFI, BsrGI, BsrI, BsrSI, BssAI, BssECI, BssHII, BssMI, BssNAI, BssNI, BssSI, BssT1I, Bst1107I, Bst2BI, Bst2UI, Bst4CI, Bst6I, BstACI, BstAFI, BstAPI, BstAUI, BstBAI, BstBI, BstC8I, BstDEI, BstDSI, BstEII, BstENI, BstF5I, BstFNI, BstH2I, BstHHI, BstKTI, BstMAI, BstMBI, BstMCI, BstMWI, BstNI, BstNSI, BstOI, BstPAI, BstPI, BstSCI, BstSFI, BstSLI, BstSNI, BstUI, BstV1I, BstV2I, BstX2I, BstXI, BstYI, BstZ17I, BstZI, Bsu15I, Bsu36I, BsuI, BsuRI, BsuTUI, BtgI, BtgZI, BtrI, BtsCI, BtsI, BtsIMutI, BtuMI, BveI, Cac8I, CaiI, CciI, CciNI, CfoI, Cfr10I, Cfr13I, Cfr42I, Cfr9I, ClaI, CpoI, CseI, CsiI, Csp6I, CspAI, CspCI, CspI, CviAII, CviJI, CviKI-1, CviQI, DdeI, DinI, DpnI, DpnII, DraI, DraIII, DrdI, DriI, DseDI, EaeI, EagI, Eam1104I, Eam1105I, EarI, EciI, Ecl136II, EclXI, Eco105I, Eco130I, Eco147I, Eco24I, Eco31I, Eco32I, Eco47I, Eco47III, Eco52I, Eco53kI, Eco57I, Eco72I, Eco81I, Eco88I, Eco91I, EcoICRI, EcoNI, EcoO109I, EcoO65I, EcoP15I, EcoRI, EcoRII, EcoRV, EcoT14I, EcoT22I, EcoT38I, EgeI, EheI, ErhI, Esp3I, FaeI, FaiI, FalI, FaqI, FatI, FauI, FauNDI, FbaI, FblI, Fnu4HI, FnuDII, FokI, FriOI, FseI, Fsp4HI, FspAI, FspBI, FspEI, FspI, GlaI, GluI, GsaI, GsuI, HaeII, HaeIII, HapII, HgaI, HhaI, Hin1I, Hin1II, Hin6I, HincII, HindII, HindIII, HinfI, HinP1I, HpaI, HpaII, HphI, Hpy166II, Hpy188I, Hpy188III, Hpy8I, Hpy99I, HpyAV, HpyCH4III, HpyCH4IV, HpyCH4V, HpyF10VI, HpyF3I, HpySE526I, Hsp92I, Hsp92II, HspAI, KasI, KflI, Kpn2I, KpnI, KroI, Ksp22I, KspAI, KspI, Kzo9I, LguI, LmnI, LpnPI, Lsp1109I, LweI, MabI, MaeI, MaeII, MaeIII, MalI, MauBI, MbiI, MboI, MboII, MfeI, MflI, MhlI, MlsI, MluCI, MluI, MluNI, Mly113I, MlyI, MmeI, MnlI, Mox20I, Mph1103I, MreI, MroI, MroNI, MroXI, MscI, MseI, MslI, Msp20I, MspA1I, MspCI, MspI, MspJI, MspR9I, MssI, MunI, Mva1269I, MvaI, MvnI, MwoI, NaeI, NarI, NciI, NcoI, NdeI, NdeII, NgoMIV, NheI, NlaIII, NlaIV, NmeAIII, NmuCI, NotI, NruI, NsbI, NsiI, NspI, NspV, OliI, PacI, PaeI, PaeR7I, PagI, PalAI, PasI, PauI, PceI, PciI, PciSI, PcsI, PctI, PdiI, PdmI, PfeI, Pfl23II, PflFI, PflMI, PfoI, PinAI, Ple19I, PleI, PluTI, PmaCI, PmeI, PmlI, PpsI, Ppu21I, PpuMI, PscI, PshAI, PshBI, PsiI, Psp124BI, Psp1406I, Psp5II, Psp6I, PspCI, PspEI, PspFI, PspGI, PspLI, PspN4I, PspOMI, PspPI, PspPPI, PspXI, PsrI, PstI, PstNI, PsuI, PsyI, PteI, PvuI, PvuII, RgaI, RigI, RruI, RsaI, RsaNI, RseI, Rsr2I, RsrII, SacI, SacII, SalI, SapI, SaqAI, SatI, Sau3AI, Sau96I, SauI, SbfI, ScaI, SchI, ScrFI, SdaI, SduI, SetI, SexAI, SfaAI, SfaNI, SfcI, SfiI, SfoI, Sfr274I, Sfr303I, SfuI, SgeI, SgfI, SgrAI, SgrBI, SgrDI, SgsI, SinI, SlaI, SmaI, SmiI, SmiMI, SmlI, SmoI, SnaBI, SpeI, SphI, SrfI, Sse8387I, Sse9I, SseBI, SsiI, SspDI, SspI, SspMI, SstI, StuI, StyD4I, StyI, SwaI, TaaI, TaiI, TaqI, TaqII, TasI, TatI, TauI, TfiI, Tru1I, Tru9I, TscAI, TseFI, TseI, Tsp45I, TspDTI, TspGWI, TspMI, TspRI, Tth111I, Van91I, Vha464I, VneI, VpaK11BI, VspI, XagI, XapI, XbaI, XceI, XcmI, XhoI, XmaI, XmaJI, XmiI, XmnI, XspI, ZraI, ZrmI, Zsp2I, Bce83I, BetI, BfiI, BinI, BsiI, BsiYI, BspLU11I, BspMII, BssKI, CauII, CfrI, CviRI, DraII, DsaI, EspI, HgiJII, Hin4II, Hpy178III, McrI, NspBII, SecI, SfeI, Tsp4CI, XhoII, XmaIII* |
| **152 REBASE commercial enzymes that cut all *P. salmonis* non-redundant sequences** |
| *AatII, BbvI, ApoI, AgsI, AjuI, AciI, BcgI, AluI, BceAI, AvaI, AlwNI, AflIII, ApaI, BglII, BsaAI, BseRI, BslFI, BsmAI, BspCNI, BspHI, BspMI, BsrI, BsrDI, BstEII, AcyI, AsuI, BalI, Bce83I, BciT130I, BetI, BfiI, BinI, BisI, BlsI, BseGI, BseMII, BseSI, BsiI, BsiYI, Bsp120I, Bsp1407I, BspMII, BssKI, BstKTI, BtgZI, BtsI, BtsIMutI, Cac8I, CauII, Cfr10I, Cfr9I, CfrI, Csp6I, CviAII, CviJI, CviRI, DdeI, DpnI, DraII, DraIII, DsaI, Eam1105I, EciI, Eco57I, EcoNI, EcoP15I, EcoRII, EcoRV, FaiI, FalI, FatI, FauI, FnuDII, FokI, GlaI, HaeII, HaeIII, HgaI, HgiJII, HhaI, Hin4II, Hin6I, HindII, HinfI, HpaII, HphI, Hpy166II, Hpy178III, Hpy188I, Hpy99I, LpnPI, MaeI, MaeII, MaeIII, MboI, MboII, MluCI, MluI, MlyI, MmeI, MnlI, MseI, MslI, MspJI, MwoI, NcoI, NlaIII, NlaIV, NruI, NspBII, NspI, OliI, PasI, PcsI, PflMI, PleI, PmaCI, PstI, RsaI, SacII, SauI, ScrFI, SduI, SecI, SetI, SexAI, SfaNI, SfeI, SgeI, SmaI, SmlI, SphI, SspI, StuI, StyI, TaiI, TaqI, TatI, TauI, TfiI, TseI, Tsp45I, Tsp4CI, TspDTI, TspGWI, TspRI, Tth111I, VspI, XbaI, XhoII, XmnI, ZraI* |
| **65 REBASE commercial enzymes that generate the same digestion pattern in all non-redundant *P. salmonis* sequences** |
| *AatII, BbvI, AjuI, BcgI, AvaI, AlwNI, BglII, BsaAI, BslFI, BsmAI, BspHI, BspMI, BsrI, BsrDI, BstEII, AcyI, AsuI, BalI, BfiI, BseGI, BseSI, BsiI, Bsp120I, Bsp1407I, BspMII, BtsI, Cfr9I, Eam1105I, EciI, Eco57I, EcoNI, EcoP15I, EcoRV, FalI, FnuDII, FokI, HaeII, HgaI, HpaII, Hpy99I, MboII, MluI, MlyI, MslI, NcoI, NruI, NspBII, OliI, PleI, PmaCI, SacII, SauI, SmaI, SphI, SspI, StuI, TatI, TseI, TspDTI, TspGWI, Tth111I, VspI, XbaI, XmnI, ZraI* |
| **13 REBASE commercial enzymes that generate different digestion patterns in all *P. salmonis* non-redundant sequences compared to other bacteria** |
| *AjuI, AsuI, BbvI, BsaAI, BseGI, Eco57I, FnuDII, FokI, HpaII, MboII, MslI, OliI, PmaCI* |

**Supplementary Table 3. Restriction enzymes with digestion patterns identical in *P. salmonis* and in different bacterial taxa**

**Supplementary Table 4. Quantitative PCR-based assays.** Real time PCR assay and Taqman probe assay.
