# Supplementary figures and images for: Genomic-Based Restriction Enzyme Selection for Specific Detection of Piscirickettsia salmonis by 16S rDNA PCR-RFLP
Source: Front Microbiol. 2016 May 9;7:643. doi: 10.3389/fmicb.2016.00643 (PMC4860512; doi:10.3389/fmicb.2016.00643)

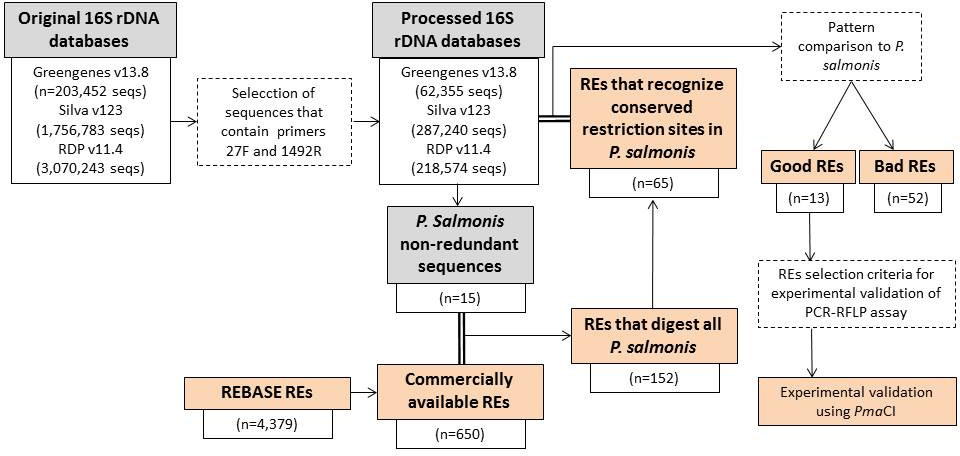

Supplement: Supplementary file 6 [file Image1.JPEG]

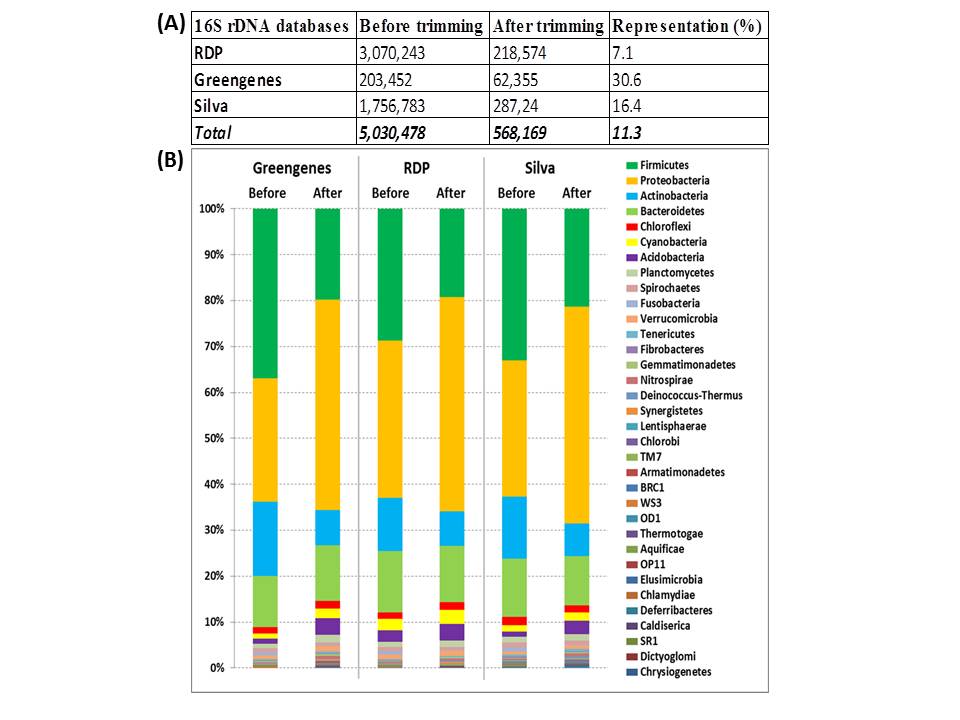

Supplement: Supplementary file 7 [file Image2.JPEG]

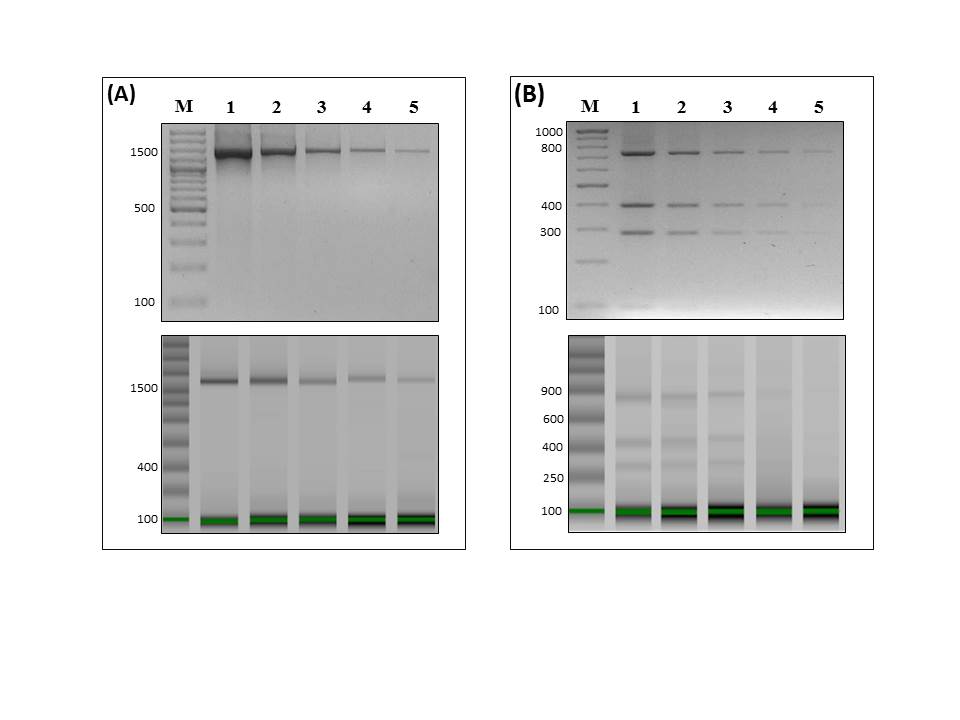

Supplement: Supplementary file 8 [file Image3.JPEG]
